# Urine CA125 and HE4 for the Detection of Ovarian Cancer in Symptomatic Women

**DOI:** 10.3390/cancers15041256

**Published:** 2023-02-16

**Authors:** Chloe E. Barr, Kelechi Njoku, Gemma L. Owens, Emma J. Crosbie

**Affiliations:** 1 Manchester Academic Health Science Centre, Department of Obstetrics and Gynaecology, St Mary’s Hospital, Manchester University NHS Foundation Trust, Manchester M13 9WL, UK; 2 Division of Cancer Sciences, Faculty of Biology, Medicine and Health, University of Manchester, Manchester M13 9WL, UK

**Keywords:** CA125, HE4, non-invasive, urine, ovarian cancer, detection

## Abstract

**Simple Summary:**

Early diagnosis of ovarian cancer is challenging due to vague symptoms and a lack of screening tools. We aimed to evaluate two simple, non-invasive urine biomarkers, CA125 and HE4, for the diagnosis of ovarian cancer in symptomatic women. Paired urine and serum samples were collected from women undergoing treatment for ovarian cancer (cases) or investigations for gynaecological symptoms (controls) and were tested for CA125 and HE4. This study found urine CA125 and HE4 levels to be higher in women with cancer compared to controls, and that a combination of urine CA125 and HE4 had a sensitivity that was similar to the currently used serum CA125 for the detection of ovarian cancer. This suggests that urine CA125 and HE4 may be useful non-invasive diagnostic tools to triage women for formal ovarian cancer investigations and could be used as point-of-care testing in the community or low resource settings.

**Abstract:**

The symptoms of ovarian cancer are vague, and current risk assessment tools such as serum CA125 and transvaginal ultrasound scan fail to reliably detect the disease early. This study aimed to evaluate urine CA125 and HE4 as diagnostic biomarkers for ovarian cancer in symptomatic women. Paired urine and serum samples were collected from women undergoing treatment for ovarian cancer (cases) or investigations for gynaecological symptoms (controls). Biomarkers were measured using an automated chemiluminescent enzyme immunoassay analyser. Standard diagnostic accuracy metrics were calculated. In total, 114 women were included, of whom 17 (15%) were diagnosed with an epithelial ovarian malignancy. Levels of urine CA125 and HE4 were significantly elevated in women with ovarian cancer compared to controls [CA125: 8.5 U/mL (IQR: 2.4–19.5) vs. 2.3 U/mL (IQR: 1.0–6.4), *p* = 0.01. HE4: 12.0 nmol/L (IQR: 10.3–23.1) vs. 6.7 nmol/L (IQR: 3.4–13.6), *p* = 0.006]. Urine CA125 and HE4 detected ovarian cancer with an AUC of 0.69 (95% CI: 0.55–0.82) and 0.71 (95% CI: 0.69–0.82), respectively (*p* = 0.73). A combination of urine CA125 and HE4 at optimal thresholds had a sensitivity of 82.4% (95% CI: 56.6–96.2) and was comparable to the sensitivity of serum CA125 [88.2% (95% CI: 63.6–98.5)]. Larger studies are required to confirm our findings, standardise urine collection, and evaluate optimal biomarker thresholds. Urine CA125 and HE4 may be useful non-invasive diagnostic tools to triage women for formal ovarian cancer investigations.

## 1. Introduction

Ovarian cancer affects 7495 women annually in the United Kingdom (UK) and has the highest mortality rate of the gynaecological malignancies [1]. Ovarian cancer lacks specific symptoms and current tests, such as serum Cancer Antigen 125 (CA125) and transvaginal ultrasound scans (TVS), lack reliability for early diagnosis and screening, leading to around two-thirds of women being diagnosed at an advanced stage (stages III and IV) [2]. Survival from ovarian cancer improves significantly when the disease is diagnosed at an early stage as over 90% of women diagnosed with stage I disease survive 5 years compared to only 25% of those with stage III [3]. A novel biomarker that can detect ovarian cancer whilst the disease burden is lower would improve clinical outcomes [4].

The symptoms of ovarian cancer are vague, have an insidious onset, and are extremely common presentations. In the UK, women with suspected ovarian cancer are recommended to undergo a serum cancer antigen 125 (CA125) and transvaginal ultrasound scanning (TVS) in the community, with a referral to secondary care if both results are abnormal or if clinical features prompt suspicion of a pelvic mass or ascites [5]. Despite these guidelines, there are commonly delays in diagnosis, with some cases taking over 12 months from presentation [6]. Reasons for delay are multifactorial and include a lack of recognition of the significance of symptoms, failure to investigate symptoms, treatment of benign causes, a lack of follow-up, and delayed referral [7]. Furthermore, whilst serum CA125 has an excellent sensitivity for advanced stage disease, it lacks the sensitivity required to diagnose ovarian cancer at an early stage. The specificity is poor, particularly in pre-menopausal women, as benign gynaecological conditions such as endometriosis can cause it to be elevated [8]. Serum human epididymis protein 4 (HE4), a promising ovarian cancer biomarker [9], is less influenced by benign gynaecological disease, with evidence suggesting it is more specific than CA125 for ovarian cancer [10,11].

Alternative sources of biomarkers, particularly urine, have gained interest over recent years. Urine is an attractive alternative to blood as it can be self-collected at home, is non-invasive, has a simpler proteome and is not under the influence of homeostatic mechanisms in the same way as blood [12]. CA125 and HE4 are detectable in urine as well as serum [13,14], with evidence suggesting that urine HE4 may be sensitive and highly specific for ovarian cancer diagnosis [15]. An accurate non-invasive biomarker that could help triage women with vague symptoms for further investigations and detect ovarian cancer whilst still at a low volume of disease could improve the survival and quality of life of women affected.

The aim of this study is to evaluate urine CA125 and HE4 as diagnostic biomarkers of ovarian cancer in symptomatic women presenting to secondary care. We hypothesised that urine CA125 and HE4 would accurately differentiate between ovarian cancer cases and controls.

## 2. Materials and Methods

### 2.1. Study Population

We recruited women who were attending St. Mary’s Hospital, Manchester, UK for the investigation of suspected gynaecological malignancy or management of presumed ovarian cancer. Women were identified from referrals to the cancer exclusion clinic and from gynaecological oncology multidisciplinary team (MDT) meetings. Women were excluded if they had previously undergone a hysterectomy or had received neo-adjuvant chemotherapy.

Women were recruited either from the cancer exclusion clinic, outpatient clinic or on the morning of their surgery. Women undergoing surgical management were recruited pre-operatively. The surgical management of suspected ovarian cancer was in line with current guidance [16], aiming for complete cytoreduction of the disease where safely possible. Women with a histological diagnosis of ovarian cancer were included as cases, and their final histology subtype, grade and FIGO 2014 staging were recorded. Women were included as controls if they had either benign gynaecological pathology or no demonstrable pathology on investigation, +/− surveillance with ultrasound, blood biomarkers, or histology.

### 2.2. Laboratory Assay Testing

Research samples were collected prior to any clinical procedures. Women were asked to produce a self-collected urine sample in a dry pot. Serum samples were taken by routine venepuncture.

Serum and urine samples were processed without fixing and stored at −80 °C in the MFT Biobank until analysis. Urine samples were centrifuged at 1000 g for 10 min to remove debris, and supernatant fractions used for CA125 and HE4 analysis. Samples were analysed for CA125 and HE4 using the Fujirebio Lumipulse^®^ G600II (Fujirebio Europe N.V., Ghent, Belgium) automated analyser, which uses a chemiluminescent enzyme immunoassay technique. In brief, this is a two-step sandwich immunoassay method. The luminescent signal generated by the final enzyme reaction is read by the analyser and reflects the level of analyte in the sample. The immunoreaction cartridges used were the Lumipulse^®^ G CA125-II and Lumipulse^®^ G HE4 (reference 292631 and 234068, respectively, Fujirebio Europe N.V., Ghent, Belgium). Analyte concentration was calculated by the analyser from the calibration curve. Calibration of the analyser for CA125 and HE4 was performed every 28 days, when a new batch of immunoreaction cartridges was used or if the control was out of range, as per the manufacturer’s advice.

Urine samples had not been previously analysed for CA125 and HE4 on the Lumipulse^®^ G600II analyser (Fujirebio Europe N.V., Ghent, Belgium), and so underwent serial dilutions to ascertain the optimal dilution for analysis and achieved good dilutional linearity. As a result of serial dilutions, urine samples were tested undiluted for CA125 and diluted to 1:100 for HE4. Samples were diluted with diluent (Lumipulse^®^ G Specimen Diluent, reference 231180, Fujirebio Europe N.V., Ghent, Belgium) as recommended by the manufacturer. Serum samples were analysed undiluted.

Samples were thawed to room temperature prior to testing and were analysed in accordance with the manufacturers protocol. Quality controls (TML 1 and 2, reference 108-20, Fujirebio, Ghent, Belgium) were run before and after each batch of patient samples. Where the analyte level was at the maximum detection limit (1000 U/L for CA125 and 1500 pmol/L for HE4), the sample was further diluted and re-tested. Urine samples were tested in triplicate and the mean taken. The median intra-assay coefficient of variation (CV) for the urinary triplicates was 15.45% (IQR: 12.64–20.66) for CA125 and 4.15% (IQR: 2.24–7.11) for HE4. The total inter-assay CV ranged from 5.60 to 7.52%.

### 2.3. Statistical Analysis

Continuous data were reported as medians with an interquartile range as they were non-parametric. Comparison between groups was performed using the Mann U Whitney test and chi squared test for continuous and categorical data, respectively. Correlations between the biomarkers and clinico-pathological features were calculated using Spearman’s rank. Due to the large quantities of HE4 in urine, levels were presented as nmol/L rather than pmol/L as used for serum HE4.

To evaluate the diagnostic performance of urine CA125 and HE4 for the detection of ovarian cancer, receiver operator characteristic (ROC) curves were constructed, and area under the curve (AUC) was calculated. Univariable and multivariable logistic regression models predicting ovarian cancer were calculated for urine CA125 and HE4 as continuous variables, both alone and in combination. As there is limited evidence in the literature regarding thresholds for urine CA125 and HE4, the optimal threshold for each biomarker was calculated from the ROC curves using the method of Liu [17]. The diagnostic accuracy of urine CA125 and HE4 was calculated using two by two cross tabulations, and the sensitivity, specificity, positive predictive value, and negative predictive value were reported. Serum CA125 was dichotomised based on the clinically used threshold of 35 IU/L [5]. The threshold for HE4 recommended in the literature is 70 pmol/L [14]; however, the chemiluminescent immunoassay analyser overestimates HE4 levels, and so the equivalent threshold of 77 pmol/L was used to ensure comparability [18].

The study was conducted in accordance with the STARD 2015 guidelines for diagnostic test reporting. A *p*-value of < 0.05 was considered significant. Data analyses were performed using STATA (StataCorp. 2015. Stata Statistical Software: Release 14. College Station, TX, USA: StataCorp LLC).

## 3. Results

### 3.1. Population Characteristics

In total, 114 participants were identified for inclusion in the final analysis. The median age and BMI of the whole group was 55 years (IQR: 51–58) and 27 kg/m^2^ (IQR: 24–33), respectively. Of these, 17 (15%) were diagnosed with an ovarian malignancy, 51 (45%) had a benign gynaecological pathology, and 46 (40%) had no demonstrable pathology (Table 1).

The histological subtypes of the ovarian malignancies included serous (6/17, 35%), mucinous (4/17, 24%), endometrioid (4/17, 24%), clear cell (2, 12%) and mixed (1/17, 6%). Around half were high grade (47%), and the majority were early-stage (FIGO stages I+II) disease (80%). The commonest benign ovarian cyst was a serous cystadenoma (5/51, 10%). There was no significant difference in age (*p* = 0.37) or BMI (*p* = 0.77) between those with ovarian cancer and those without.

### 3.2. Summary of Urine CA125 Levels

The median urine CA125 of the total population was 3.3 U/mL (IQR: 1.1–10.0) and values ranged from 0.3 to1607.0 U/mL. Levels of CA125 were found to be significantly higher in serum samples [14.3 U/mL (IQR: 10.1–25.1)] compared to urine samples (*p* < 0.001). There was no significant correlation between urine CA125 and age (Spearman’s Rho −0.05, *p* = 0.56) or BMI (Spearman’s Rho 0.08, *p* = 0.39); however, there was weak evidence of correlations with serum CA125 (Spearman’s Rho 0.26, *p* = 0.003), serum HE4 (Spearman’s Rho 0.31, *p* < 0.001) and urine HE4 (Spearman’s Rho 0.33, *p* < 0.001). No significant difference was found in median urine CA125 levels in those aged over 55 years [2.8 U/mL (IQR: 1.0–7.1)] compared to those under 55 years [4.0 U/mL (IQR: 1.4–11.2) *p* = 0.38]. In women with a BMI ≥30 kg/m^2^, the median urine CA125 was 3.8 U/mL (IQR: 1.4–9.0) compared to 2.7 U/mL (IQR: 1.1–10.6) in women with a BMI < 30 kg/m^2^ (*p* = 0.39).

**Figure 1:**
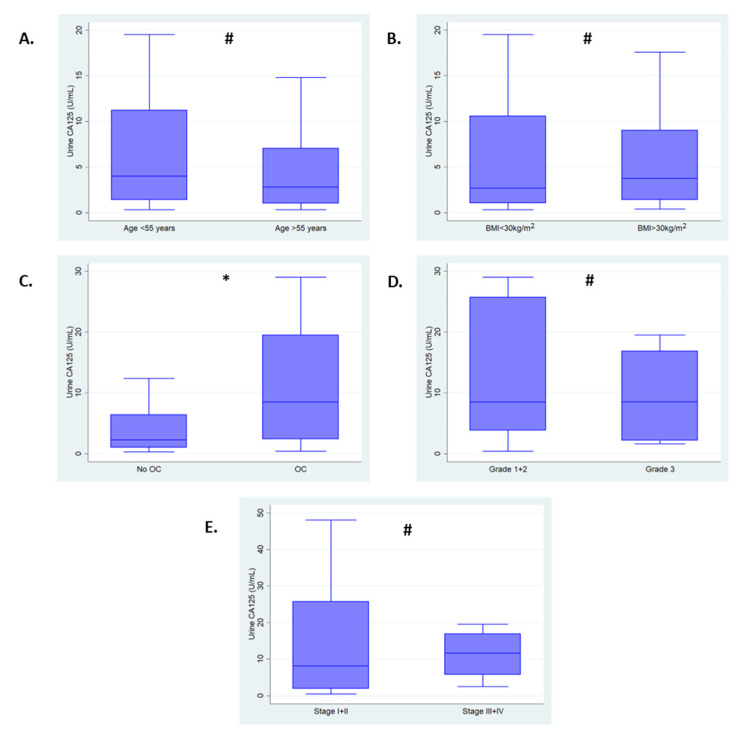
Box plots of urine CA125 (U/mL) levels and clinico-pathological features. (**A**) Age (<55 years vs. >55 years). (**B**) BMI (<30 kg/m^2^ vs. >30 kg/m^2^). (**C**) Disease status (no ovarian cancer vs. ovarian cancer). (**D**) Grade (low grade vs. high grade). (**E**) FIGO Stage (early stage vs. late stage). Significance calculated using Mann-U Whitney test * *p*-value < 0.05. # *p*-value ≥ 0.05. Outliers excluded from plots. OC—ovarian cancer.

Figure 1 shows the association of urine CA125 with clinico-pathological features. Median urine CA125 levels were found to be significantly elevated in women with ovarian cancer [8.5 U/mL (IQR: 2.4–19.5)] compared to those without [2.3 U/mL (IQR: 1.0–6.4), *p* = 0.01]. There was no evidence of an association between urine CA125 levels and cancer grade (*p* = 0.85). Whilst median urine CA125 levels were observed to be higher in those with advanced stage disease [11.6 U/mL (IQR: 5.7–16.9)] compared to those with early stage disease [8.1 U/mL (IQR: 1.9–25.7)], this did not reach statistical significance (*p* = 0.65), likely due to small numbers.

### 3.3. Summary of Urine HE4 Levels

Unlike CA125, the HE4 concentration was found to be significantly higher in urine compared to serum [79.0 pmol/L (IQR: 63.6–115.2) vs. 8415.0 pmol/L (IQR: 3825–14,338), *p* < 0.001], necessitating the conversion of values from pmol/L to nmol/L for simplicity. The values of urine HE4 ranges from 0.6 nmol/L to 1717.0 nmol/L, with a median of 8.41 nmol/L (IQR: 3.84–14.33). Whilst urine HE4 correlated with serum CA125 (Spearman’s Rho 0.22, *p* = 0.02), serum HE4 (Spearman’s Rho 0.40, *p* < 0.001) and urine CA125 (Spearman’s Rho 0.33, *p* < 0.001), no significant correlation was seen with age (Spearman’s Rho −0.118, *p* = 0.21) or BMI (Spearman’s Rho −0.03, *p* = 0.72). In women over 55 years, median urine HE4 was 6.7 nmol/L (IQR: 3.4–11.8) compared to 10.2 nmol/L (IQR: 4.1–21.1) in women under 55 years (*p* = 0.07). There was no significant difference in median urine HE4 levels between women with a BMI < 30 kg/m2 [7.9 nmol/L (IQR: 4.0–15.6)] and those with a BMI ≥ 30 kg/m^2^ [9.1 nmol/L (IQR: 2.8–12.9), *p* = 0.64].

**Figure 2:**
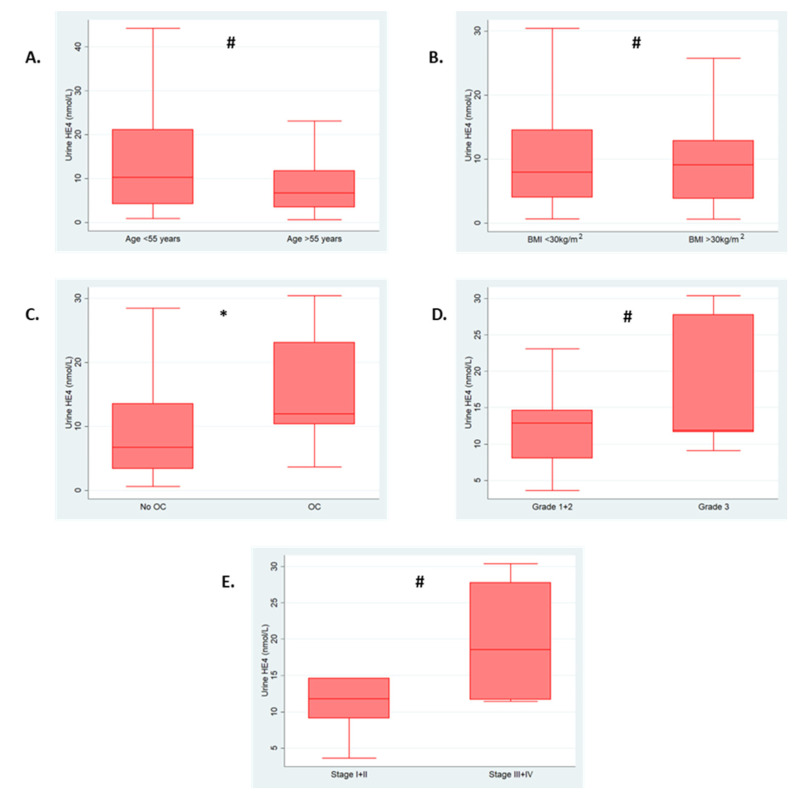
Box plots of urine HE4 (nmol/L) levels and clinico-pathological features. (**A**) Age (<55 years vs. >55 years). (**B**) BMI (<30 kg/m^2^ vs. >30 kg/m^2^). (**C**) Disease status (no ovarian cancer vs. ovarian cancer). (**D**) Grade (low grade vs. high grade). (**E**) FIGO Stage (early stage vs. late stage). Significance calculated using Mann-U Whitney test * *p*-value < 0.05. # *p*-value ≥ 0.05. Outliers excluded from plots. OC—ovarian cancer.

Urine HE4 levels were significantly higher in women with ovarian cancer [12.0 nmol/L (IQR: 10.3–23.1)] compared to those without [6.7 nmol/L (IQR: 3.4–13.6), *p* = 0.006]. There was no evidence of a difference between those with low- and high-grade disease [12.9 nmol/L (IQR: 8.01–14.7) vs. 11.9 nmol/L (IQR: 11.6–27.8), *p* = 0.39], or those with early- and advanced-stage disease [11.8 nmol/L (IQR: 9.1–14.7) vs. 18.5 nmol/L (IQR: 11.7–27.8), *p* = 0.31]. Figure 2 shows the association between urine HE4 levels and clinico-pathological features.

### 3.4. Summary of Serum CA125 and HE4

No significant correlation was observed between serum CA125 and age (Spearman’s Rho 0.09, *p* = 0.31) or BMI (Spearman’s Rho 0.04, *p* = 0.65). Levels of serum CA125 were significantly elevated in women with ovarian cancer [233.4 U/mL (IQR: 92.2–424.1)] compared to those without [13.6 U/mL (IQR: 9.3–19.0)]; however, there was no significant difference in biomarker levels between low- and high-grade disease [151.5 U/mL (IQR: 89.9–424.1) vs. 262.9 U/mL (IQR: 154.9–14,407.7), *p* = 0.25] or early- and advanced-stage disease [156.3 U/mL (IQR: 89.9–355.4) vs. 14,356.5 U/mL (IQR: 203.2–30,305.0), *p* = 0.11].

There was no significant correlation between serum HE4 and age (Spearman’s Rho 0.16, *p* = 0.08), or BMI (Spearman’s Rho −0.019, *p* = 0.84). Serum HE4 levels were significantly elevated in women with ovarian cancer compared to those without [213.6 pmol/L (IQR: 149.0–295.8) vs. 75.7 pmol/L (IQR: 61.4–93.5), *p* < 0.001]. In addition, serum HE4 levels were elevated in those with high grade disease [291.6 pmol/L (IQR: 258.5–620.2)] compared to those with low grade disease [149.0 pmol/L (IQR: 88.6–208.0), *p* = 0.009]. There was no significant difference in the serum levels of HE4 between those with early stage disease and those with advanced stage disease [185.9 pmol/L (IQR: 132.6–235.5) vs. 291.6 pmol/L (IQR: 284.4–479.6), *p* = 0.09].

### 3.5. Diagnostic Accuracy of Urine CA125 and HE4

**Figure 3:**
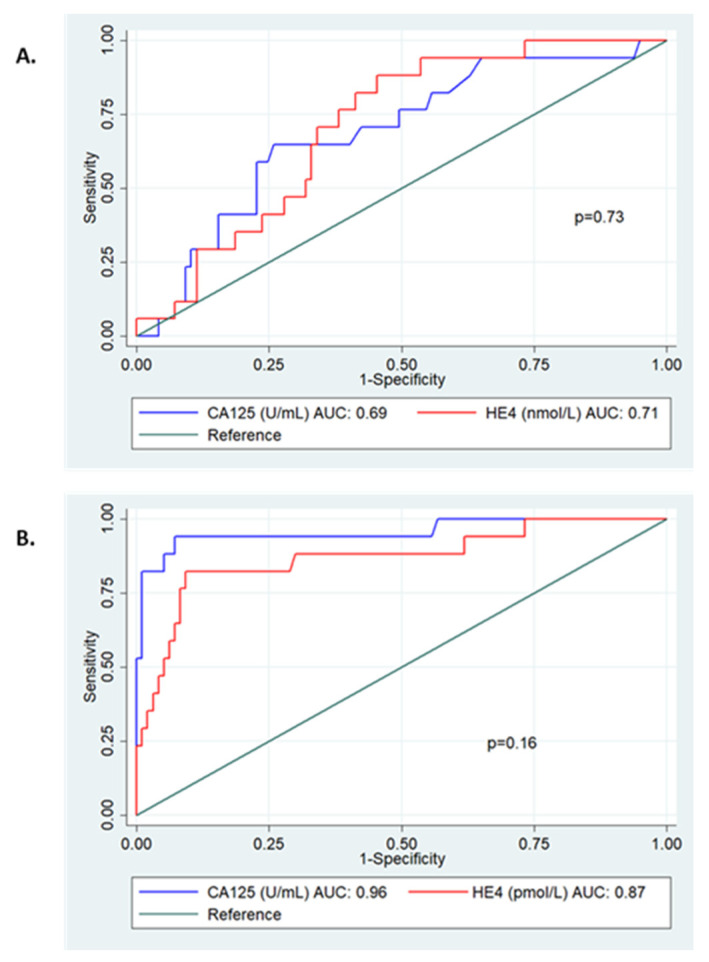
ROC curve analysis of serum and urine CA125 and HE4 for the detection of epithelial ovarian cancer. (**A**) Urine CA125 and HE4. CA125: AUC 0.69 (95% CI 0.55–0.82), HE4: AUC 0.71 (95% CI 0.60–0.82), *p* = 0.73. (**B**) Serum CA125 and HE4. CA125: AUC 0.96 (95% CI 0.89–1.00), HE4: AUC 0.87 (95% CI 0.76–0.98), *p* = 0.18.

Urine CA125 and HE4 were able to predict epithelial ovarian cancer with a model AUC of 0.69 (95% CI: 0.55–0.82, *p* = 0.01) and 0.71 (95% CI: 0.60–0.82, *p* = 0.006), respectively (Figure 3). There was no significant difference in performance (*p* = 0.73). When combined, urine CA125 did not significantly add to urine HE4 alone to predict ovarian cancer [model AUC 0.71 (95% CI: 0.60–0.82), *p* = 0.06].

The optimal diagnostic cut off was 6.2 U/mL for urine CA125, with an AUC of 0.69 and 9.1 nmol/L for urine HE4, with an AUC of 0.71. Of those with ovarian cancer, 11/17 (65%) had a urine CA125 ≥ 6.2 U/mL compared to 25/97 (26%) of those without (*p* = 0.001). Urine HE4 was positive in 12/17 (71%) of women with ovarian cancer compared to 40/97 (41%) of those without (*p* = 0.03) (Table 2).

Table 3 summaries the diagnostic accuracy of serum and urine CA125 and HE4 at their optimal thresholds. Urine HE4 had a superior sensitivity [70.6% (95% CI: 44.0–89.7)] compared to urine CA125 [64.7% (95% CI: 38.3–85.8)] but a worse specificity. Combining the urine markers using a strategy where either was positive improved the sensitivity to 82.4% (95% CI: 56.6–96.2), making the combined urine test almost as sensitive as serum CA125 [88.2% (95% CI: 63.6–98.5)]. In our cohort, the combined urine test missed one more ovarian cancer (true positive 14/17) compared to serum CA125 (true positive 15/17); however, the poor specificity would lead to increased numbers of false positives. The combination of urine biomarkers where either was positive had a positive and negative likelihood ratio for the detection of ovarian cancer of 1.51 (95% CI: 1.13–2.00) and 0.39 (95% CI: 0.14–1.11), respectively, whereas serum CA125 at a threshold of 35 U/mL had positive and negative likelihood ratios of 14.3 (95% CI: 6.45–31.6) and 0.13 (95% CI: 0.03–0.46), respectively.

Urine CA125 and HE4 were able to detect early stage disease (FIGO I+II) with an AUC 0.67 (95% CI: 0.51–0.83) and AUC 0.68 (95% CI: 0.56–0.81), respectively (Figure S1). Combining the urine biomarkers where either was positive had a better sensitivity than either marker alone [76.9% (95% CI: 46.2–95.0)], but with an observed cost to specificity (Table S1).

## 4. Discussion

### 4.1. Main Finding

In this study, we evaluated urine CA125 and HE4 for the detection of ovarian cancer. Urine CA125 and HE4 levels were significantly higher in women with ovarian cancer compared to those without and were able to detect disease with moderate accuracy. At optimal thresholds, a combination of urine CA125 and HE4 where either was positive had a sensitivity similar to that of serum CA125 and, in our cohort, missed one additional cancer compared with serum CA125. If confirmed in larger studies, a combination of urine CA125 and HE4 could be of use as a non-invasive point-of-care triage tool to identify those requiring formal investigation for ovarian cancer; however, our numbers are small and confidence intervals wide. Large prospective studies are needed to validate our findings and optimise urine biomarker thresholds.

### 4.2. Strengths and Limitations

To our knowledge, this is the first study investigating the combined diagnostic performance of urine CA125 and HE4 for the detection of ovarian cancer. Major strengths of our study include its prospective design and that we had matched urine and serum samples, allowing direct comparison of diagnostic performance. Our study is limited by the small sample size, and we recognise that there are a small number of ovarian cancer cases. The number of ovarian cancer cases in our study population is lower (15%) than those in many other diagnostic biomarker studies in the literature, which often report a prevalence of between 20 to 50% [19]. Our study therefore represents a much more realistic sample of the numbers of ovarian cancer cases in symptomatic women in secondary care, which are estimated to be around 10% [20], which is an important consideration due to the variation in diagnostic performance of biomarkers in different populations [21]. Furthermore, our sample may not be fully representative of other ovarian cancer populations as it comprised of a high number of women with early-stage disease and non-serous subtypes. As such, how the urine biomarkers might perform in a population with a lower prevalence of early-stage disease is unknown. Nevertheless, it is promising that urine CA125 and HE4 in combination has a comparable sensitivity to serum CA125 in a population where 80% were diagnosed at an early-stage of the disease. Larger studies that include the full spectrum of histological subtypes and ages are required to establish the true diagnostic accuracy of urine CA125 and HE4. Urine is a source of a large number of different proteins, and the levels are influenced by a number of factors, such as renal function, medications, time of day, and fluid consumption. We have been unable to account for these confounders within our study, and further work is needed to standardise urine collection and normalise urine CA125 and HE4 levels to enable a more reliable assessment of accuracy. Furthermore, our control group includes women attending with gynaecological symptoms, giving a more accurate idea of specificity; however, we are unable to comment on how the biomarkers might perform in a healthy or symptomatic primary care population, and the factors that might influence their levels.

### 4.3. Comparison with the Current Literature

Urine has gained increasing interest as a source of biomarkers and is cheap, acceptable to patients, and less influenced by homeostatic mechanisms than serum. Despite this, there are few studies investigating urine CA125 and HE4 for the detection of ovarian cancer, and none evaluating the two markers in combination. Only two studies report on the diagnostic accuracy of urine CA125. Moore et al. reported a disappointing sensitivity of less than 40% in 67 women with ovarian cancer and 166 women with benign adnexal masses [14]. Tay et al. reported a sensitivity of 88.9% in 105 women with adnexal masses, 10 of whom had ovarian cancer [22]. HE4 is the most studied urine protein biomarker for ovarian cancer and has been investigated both alone and in combination with other proteins such as mesothelin [23] and in multi-biomarker panels [24] with promising results. In a meta-analysis by Jia et al., which included 413 cases and 573 controls pooled from seven studies, urine HE4 had an AUC of 0.94, a sensitivity of 76% and a specificity of 92% [15]. Two further small case-control studies have also reported urine HE4 sensitivities of more than 80% for the detection of ovarian cancer [25]. In our study, urine CA125 and HE4 individually demonstrated poorer diagnostic accuracy compared to those reported in the literature. This is likely due to our small numbers and case mix that included disproportionate numbers of women with early stage, non-serous disease. There is much heterogeneity amongst published studies, with significant differences in the histological subtypes and stages included. Few studies report urine sampling methods, and those that do differ in collection times, fasting status, and normalisation of protein levels [25], challenging interstudy comparability.

### 4.4. Clinical and Research Implications

Serum biomarkers have been much more extensively studied, with CA125 in routine clinical use for the diagnosis of ovarian cancer worldwide. Serum HE4 is a promising diagnostic biomarker and has been evaluated both alone and as part of diagnostic algorithms, of which, ROMA seems to be superior [9]. No studies have evaluated urine markers and serum markers within the same population.

There is considerable interest in developing alternative biofluids for disease detection. Advances in technology mean that biofluids with lower biomarker concentrations can now be exploited for clinical use. Urine in particular is an attractive biofluid, as it is cheap, readily available, and non-invasive. Our study suggests that a combination of urine biomarkers may be a promising tool for ovarian cancer detection and, at optimal thresholds, may have a comparable sensitivity to serum CA125. The relatively poor specificity of the combined urine biomarker test (40.2%) is offset by its high sensitivity (82.4%), since a triage tool must first seek to not falsely reassure. Whilst our study suggests a comparable accuracy to CA125, urine biomarkers are not intended to replace CA125 and imaging in secondary care; however, if our findings were confirmed in larger populations, urine biomarkers may have a role in triaging symptomatic women for additional testing in the community and in low-resource settings, or as a home-based monitoring tool for women at high risk of developing ovarian cancer.

Diagnostic delays in primary care are due to vague symptoms, a low incidence of disease, a lack of recognition of the significance of symptoms, and a failure to investigate and follow up on those who are symptomatic [7]. A urine-based point-of-care test could identify those who require formal investigation, increase investigation of vague symptoms, and provide a quick and easy way of following up with women in the community who have ongoing symptoms and concerns. It would allow women with a negative test to be reassured promptly and alternative diagnoses explored. Home-based testing could potentially reduce visits to healthcare providers for those undergoing screening, surveillance or monitoring. For women who are carriers of *BRCA* pathogenic variants and at high risk of developing ovarian cancer, a risk-reducing bilateral salpingo-oophorectomy is recommended following the completion of childbearing [16]. However, despite this being the most effective option for risk reduction, many opt not to undergo surgery due to a desire to retain fertility and the impact of surgical menopause on long-term quality of life [26]. Surveillance strategies are being assessed using longitudinal biomarker data [27,28], and urine-based biomarkers offer the unique opportunity for cheap and convenient home-based testing for surveillance of those at high risk of ovarian cancer. Simple, non-invasive, painless, cost-effective and convenient diagnostic tests were a priority for both patients and clinicians in the James Lind Alliance Priority Setting Partnership for Detecting Cancer Early [29], and urine certainly fulfils these criteria.

In low-resource healthcare settings and in areas of healthcare inequalities, diagnostic urine biomarkers offer an exciting opportunity to improve ovarian cancer detection. Although mortality rates of ovarian cancer are highest in high income countries, reflecting more incident cases, the mortality/incidence ratios are greatest in low-income countries, due to availability, cost and access to diagnostic investigations [30]. Even within high-income countries, healthcare inequalities lead to disparities in survival outcomes for ovarian cancer, as a result of delayed, restricted and limited access to healthcare [31], with evidence suggesting those without private healthcare insurance are more likely to present with advanced-stage disease [32]. Diagnostic urine biomarkers have huge potential to improve detection rates of ovarian cancer in these populations, as they are cheap, easy to obtain, and can be done remotely. Urine sampling lends itself to self-collection at home for immediate testing, using lateral flow technology or postal return to the laboratory. This offers a significant advantage over blood tests, which require travel to clinics, resulting in additional healthcare costs, travel costs, and loss of earnings through time off work.

This proof-of-concept study, comparing the combination of urine CA125 and HE4 with serum CA125, is the first to be reported, and an important preliminary step in biomarker development, demonstrating the ability of urine CA125 and HE4 to detect ovarian cancer cases from symptomatic controls, the majority of whom had early-stage disease. Biomarker development requires several phases prior to clinical translation, including discovery, analytical validation, clinical validation and clinical utility. These phases have been outlined by the Early Detection Research Network (ERDN) [33]. Our study falls under the early phases of biomarker development, providing evidence of clinical performance and optimal thresholds, which require validation in larger prospective secondary and primary care populations to demonstrate true clinical utility. Studies evaluating HE4 as a diagnostic biomarker demonstrate significant heterogeneity in design, study population, HE4 assay methodology, and thresholds for positivity. Several studies have demonstrated differences in the median serum HE4 levels, depending on the immuno-assay method used, and the same is likely to be true for urine HE4 [18,34]. The HE4 protein has five different isoforms (V0–V4), containing differing WAP terminal domains [35]. The HE4 protein variants exist in varying amounts in both normal tissue and malignant tissue, with work by Hellstrom et al. demonstrating that ovarian cancers express different HE4 epitopes, defined by four separate monoclonal antibodies with distinctive WAP terminal domain specificities [36]. Differences in HE4 variants expressed and the antibody binding specificity of the assay used will influence measured levels of HE4. It is unknown if urine HE4 protein isoforms differ significantly to those in the serum. More work is required to evaluate the assays for use in urine and harmonise different immuno-assay techniques.

## 5. Conclusions

The combination of urine CA125 and HE4 using a strategy where either was positive, shows promise for the detection of ovarian cancer, with a sensitivity comparable to that of serum CA125. Urine biomarkers are cheap and acceptable non-invasive tests, and could be a useful point-of-care tool to triage symptomatic women, or as a remote monitoring tool for women at high risk of ovarian cancer, identifying those requiring further investigations. Larger studies to confirm our findings and optimise urine collection and biomarkers thresholds are required.

## Figures and Tables

**Table 1:** Clinico-pathological characteristics of the population.

Characteristic	Number (%)
a. OC, *n* = 17 (15%)	
**Age (years)**	
Median (IQR)	58 (52–68)
**BMI (kg/m^2^)**	
Median (IQR)	29 (24–31)
**Histological subtype (*n*,%)**	
Serous	6 (35)
Mucinous	4 (24)
Endometrioid	4 (24)
Clear Cell	2 (12)
Carcinosarcoma	0 (0)
Mixed	1 (6)
**Grade (*n*,%)**	
1	6 (35)
2	3 (18)
3	8 (47)
**FIGO Stage (*n*,%)**	
I	10 (59)
II	3 (18)
III	3 (18)
IV	1 (5)
**Complete cytoreduction (*n*,%)**	
Yes	13 (76)
No	2 (12)
Missing	2 (12)
**Adjuvant Therapy (*n*,%)**	
No	4 (23)
Yes	10 (59)
Unknown	3 (18)
**b. No OC**, *n =* 97 (85%)	
**Age (years)**	
Median (IQR)	54 (51–58)
**BMI (kg/m^2^)**	
Median (IQR)	27 (24–33)
**Final diagnosis**	
**No demonstrable pathology**	**46 (47)**
**Benign**	**51 (53)**
Atrophy	13 (25)
Simple cyst	1 (2)
Serous cystadenoma	5 (10)
Mucinous cystadenoma	2 (4)
Fibroma	1 (2)
Dermoid	1 (2)
Angioleiomayata	1 (2)
Other benign cyst	2 (4)
Fibroid	9 (19)
Endo-cervical polyp	14 (28)
Prolapse	1 (2)

OC—ovarian cancer. BMI—body mass index. *n*-number. IQR—interquartile range.

**Table 2:** Two-by-two cross-tabulations of serum and urine CA125 and HE4 and ovarian cancer.

	No OC, *n* (%)	OC, *n* (%)	*p*-Value
**Urine CA125 (U/mL)**			
<6.2	72 (74)	6 (35)	0.001
≥6.2	25 (26)	11 (65)	
**Urine HE4 (nmol/L)**			
<9.1	57 (59)	5 (29)	0.03
≥9.1	40 (41)	12 (71)	
**Urine Combined**			
Negative	44 (45)	3 (18)	0.03
Positive *	53 (55)	14 (82)	
**Serum CA125 (U/mL)**			
<35	91 (94)	2 (12)	<0.001
≥35	6 (6)	15 (88)	
**Serum HE4 (pmol/L)**			0.002
<77	51 (53)	2 (12)	
≥77	46 (47)	15 (88)	

* either positive. OC—ovarian cancer. *n*—number. *p*-value calculated using chi-squared analysis.

**Table 3:** Diagnostic performance of serum and urine CA125 and HE4 for the detection of epithelial ovarian cancer at specified thresholds.

Biomarker	Sensitivity, % (95%CI)	Specificity, % (95%CI)	PPV, % (95%CI)	NPV, % (95%CI)	Positive LR, % (95%CI)	Negative LR, % (95%CI)
Urine CA125 (≥6.2 U/mL)	64.7 (38.2–85.8)	74.2 (64.3–82.6)	30.6 (16.3–48.1)	92.3 (84.0–97.1)	2.51 (1.54–4.09)	0.48 (0.25–0.92)
Urine HE4 (≥9.1 nmol/L)	70.6 (44.0–89.7)	58.8 (48.3–68.7)	23.1 (12.5–36.8)	91.9 (82.2–97.3)	1.71 (1.16–2.52)	0.50 (0.24–1.06)
Urine Combined *	82.4 (56.6–96.2)	40.2 (30.4–50.7)	19.4 (11.1–30.5)	92.9 (80.5–98.5)	1.51 (1.13–2.00)	0.39 (0.14–1.11)
Serum CA125 (≥35 U/mL)	88.2 (63.6–98.5)	93.8 (87.0–97.7)	71.4 (47.8–88.7)	97.8 (92.4–99.7)	14.3 (6.45–31.6)	0.13 (0.03–0.46)
Serum HE4 (≥77 pmol/L)	88.2 (63.6–98.5)	52.6 (42.2–62.8)	24.6 (14.5–37.3)	96.2 (87.0–99.5)	1.86 (1.42–2.44)	0.22 (0.06–0.83)

* either positive. CI—confidence interval. PPV—positive predictive value. NPV—negative predictive value. LR—likelihood ratio.

## Data Availability

Fully anonymised data are available on reasonable request to the corresponding author.

## Supplementary Materials

The following supporting information can be downloaded at: https://www.mdpi.com/article/10.3390/cancers15041256/s1, Figure S1: ROC curve analysis of serum and urogenital biomarkers for the detection of early- and late-stage epithelial ovarian cancer. A- Early-stage disease. Urine. CA125: AUC 0.67 (95% CI: 0.51–0.83), HE4: AUC 0.68 (95% CI: 0.56–0.81), *p* = 0.81. Serum. CA125: AUC 0.94 (95% CI: 0.86–1.00), HE4: AUC 0.84 (95% CI: 0.70–0.97), *p* = 0.19. B- Late-stage disease. Urine. CA125: AUC 0.76 (95% CI: 0.57–0.94), HE4: AUC 0.79 (95% CI: 0.64–0.94). *p* = 0.62. Serum. CA125: AUC 0.99 (95% CI: 0.99–1.00), HE4: AUC 0.98 (95% CI: 0.96–1.00). *p* = 0.25; Table S1: Diagnostic accuracy of serum and urine CA125 and HE4 for the detection of early-stage disease (*n* = 110, OC = 13 (12%)).
