# Supplementary material for: Urine CA125 and HE4 for the Detection of Ovarian Cancer in Symptomatic Women
Source: Cancers (Basel). 2023 Feb 16;15(4):1256. doi: 10.3390/cancers15041256 (PMC9953976; doi:10.3390/cancers15041256)
Supplement: Supplementary file 1 [file cancers-15-01256-s001.zip › cancers-2181899-supplementary.pdf]

cancers

MDPI

Article

Urine CA125 and HE4 for the Detection of Ovarian Cancer in Symptomatic Women—Supplementary materials

Chloe E. Barr, Kelechi Njoku, Gemma L. Owens, Emma J. Crosbie

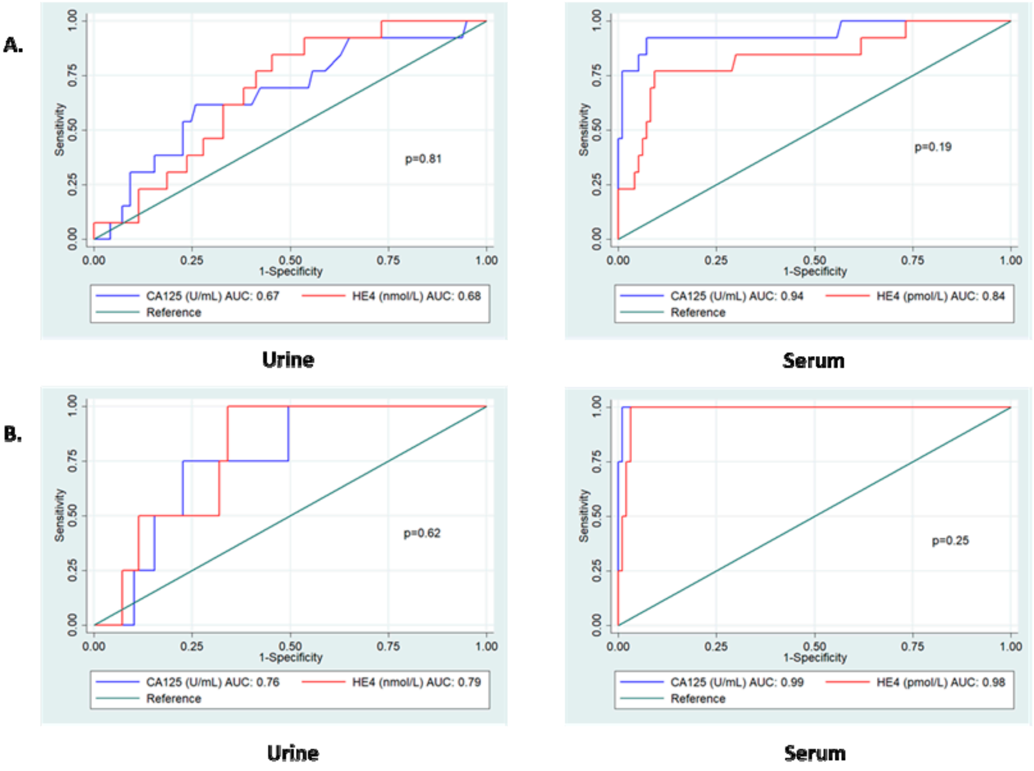

Figure S1- ROC Curve analysis of serum and urogenital biomarkers for the detection of early and late stage epithelial ovarian cancer. A- Early stage disease. Urine. CA125 AUC 0.67 (95%CI: 0.51-0.83), HE4 AUC 0.68 (95%CI: 0.56-0.81), p=0.81. Serum. CA125 AUC 0.94 (95%CI: 0.86-1.00), HE4 AUC 0.84 (95%CI: 0.70-0.97), p=0.19. B- Late stage disease. Urine. CA125 AUC 0.76 (95%CI: 0.57-0.94), HE4 AUC 0.79 (95%CI: 0.64-0.94). p=0.62. Serum. CA125 AUC 0.99 (95%CI: 0.99-1.00), HE4 AUC 0.98 (95%CI: 0.96-1.00). p=0.25.

Table S1. Diagnostic accuracy of serum and urine CA125 and HE4 for the detection of early stage disease (n=110, OC=13 (12%)).

	Sensitivity % (95%CI)	Specificity % (95%C)	PPV % (95%CI)	NPV % (95%CI)
Urine CA125 (≥6.15U/mL)	61.5 (31.6-86.1)	74.2 (64.3-82.6)	24.2 (11.1-42.3)	93.5 (85.5-97.9)
Urine HE4 (≥9.1nmol/L)	61.5 (31.6-86.1)	58.8 (48.1-68.7)	16.7 (7.48-30.2)	91.9 (82.2-97.3)
Urine Combined*	76.9 (46.2-95.0)	45.4 (35.2-55.8)	15.9 (7.88-27.3)	93.6 (82.5-98.7)
Serum CA125 (≥35U/mL)	84.6 (54.6-98.1)	93.8 (87.0-97.7)	64.7 (38.3-85.8)	97.8 (92.4-99.7)
Serum HE4 (≥77pmol/L)	84.6 (54.6-98.1)	52.6 (42.2-62.8)	19.3 (10.0-31.9)	96.2 (87.0-99.5)

*either positive. CI- confidence interval. PPV- positive predictive value. NPV- negative predictive value. LR- likelihood ratio
